# Clinical Association of CD44 Expression with Proliferative Activity and Apoptotic State in Egyptian Patients Suffering from Ulcerative Colitis and Colorectal Carcinoma

**DOI:** 10.31557/APJCP.2021.22.11.3577

**Published:** 2021-11

**Authors:** Hoda El-Emshaty, Doaa Hassan, Mohamed El-Hemaly, Hisham Ismail

**Affiliations:** 1 *Gastrointestinal Surgery Center, Faculty of Medicine, Mansoura University, Mansoura, Egypt. *; 2 *Biochemistry Division, Department of Chemistry Faculty of Science, Minia University, Minia, Egypt. *

**Keywords:** CD44, DNA cell cycle, apoptosis, flow cytometry, Egyptian, colorectal cancer

## Abstract

**Background and aim:**

Cancer stem cell markers were thoroughly investigated as a promising strategy for the prediction of patient outcome and therapeutic response. The prospective role of CD44 cell adhesion molecule in tumorigenic potential and its association with the proliferative activity and apoptotic status of Egyptian patients with ulcerative colitis (UC) and colorectal cancer (CRC) were investigated in this study.

**Material and method:**

Flow cytometric analyses of CD44, DNA cell cycle, and apoptosis identified by Annexin V/PI were performed on colonic tissue specimens obtained from 44 CRC patients, 36 UC patients, and 30 controls.

**Results:**

The CRC patients showed overexpression of CD44 marker (p < 0.0001) in comparison with UC and control groups. Regression analysis identified CD44 marker as an independent predictor for tumor staging and grading (p < 0.0001) of CRC patients. The CD44 expression was positively correlated with tumor stage (r = 0.656), tumor grade (r = 0.645), and the proliferative activity of DNA cell cycle (S phase, r = 0.396). However, CD44 expression was negatively correlated with early apoptosis (r = - 0.525).

**Conclusion:**

According to our findings, there was a significant and positive association between CD44 dysregulated expression and S phase of DNA cell cycle but a negative association with early apoptosis in CRC patients, suggesting CD44 role in apoptosis suppression reducing the tumor growth reserve.

## Introduction

Colorectal cancer (CRC) causes a significant burden on healthcare systems worldwide, which is associated with high morbidity and mortality rates (Ji et al., 2021). The risk of CRC is higher in patients with inflammatory bowel disease (IBD) (Fantini and Guadagni, 2021). There is a number of diagnostic strategies and therapeutic methods to treat CRC. Nevertheless, the overall survival rate of patients with CRC remains low due to tumor recurrence and metastasis (Lee et al., 2007; Sharkas et al., 2017). Hence, there is an urgent need to fully understand the molecular mechanisms regulating CRC initiation and progression to develop an effective therapeutic approach (Miao et al., 2018). CRC is a common disease that is usually detected at an advanced stage because it is mostly asymptomatic and appropriate serologic biomarkers have not been established at early stages (Tagi et al., 2010). The discovery of colorectal cancer stem cells (CSCs) highlighted the existence of intratumoral heterogeneity, revealing the presence of tumor cells expressing markers characteristic of immature cells and with increased abilities to resist chemotherapy and to seed secondary tumors (O’Brien et al., 2007; Wang et al., 2012). The cell surface proteins CD133, CD24 and CD44 are putative markers for CSC populations in colon cancer, which are associated with aggressive cancer types and poor prognosis (Sahlberg et al., 2014). Indeed, CD44 is a major marker for CSC, which is characterized by self-renewal capacity, epithelial-mesenchymal transition, and treatment resistance (Zöller et al., 2011). CD44, a complex transmembrane glycoprotein, exists in multiple molecular forms, namely the standard isoform CD44s and CD44 variant isoforms. CD44 is involved in the regulation of diverse vital signaling pathways modulating cancer proliferation, invasion, metastasis, and therapy-resistance (Xu et al., 2020). It is scientifically and clinically important for a better understanding of the intermediate events that predispose patients to neoplastic progression. Dysplasia observed in biopsies taken during colonoscopy of patients with IBD e.g. ulcerative colitis is a premalignant feature, which deserves further clinical and laboratory management (Haggitt, 1990). Many neoplasms generate DNA-flow cytometry histograms differing from the normal in having more than one peak with a different DNA content. The term aneuploidy is being used to describe these populations (Quinn and Wright, 1990; Tripathi et al., 2008). The existence of aneuploidy is considered a definitive marker for the presence of tumor (Darzynkiewicz et al., 2010). Moreover, it is correlated with poor prognosis and early recurrence following surgery (Tripathi et al., 2008). Analysis of DNA content reveals cell ploidy, providing information on cell position in the cell cycle and subsequently allowing one to estimate the frequency of apoptotic cells (Darzynkiewicz et al., 2010). The frequency of such apoptotic cells can be estimated by identifying cells with fractional DNA content (DI < 1.0), which is often defined as “Sub G1” cell population (Tabll and Ismail, 2011). Apoptosis is a crucial tumor suppression mechanism within the body because it contributes to the elimination of cells that have extensive DNA damage and the potentiality to develop to cancer (Atkim-Smith et al., 2015). However, defects in apoptosis influence the effectiveness of conventional therapies that mainly exert their effect by inducing apoptosis (Ricci and Zong, 2006). Therefore, the detection of appropriate biomarkers, such as CD44, might be helpful in predicting CRC at early stages. Thus, the aim of this study was to investigate the prospective role of CD44 expression in tumorigenesis and its association with the proliferative activity and apoptotic status of CRC and UC in Egyptian patients.

## Materials and Methods

*Study samples*

A total of 110 individuals who were admitted at Gastrointestinal Surgery Center (GISC), Egypt, from November 2018 to January 2020 were included in this prospective study. The participants included 44 patients with CRC (20 men and 24 women), and 36 patients with UC (18 men and 18 women), 30 individuals (12 men and 18 women) as non-disease controls (NDC). The mean age of the participants was 51.9 ± 14.77 years old in the CRC group, 40.28 ± 12.53 years old in the UC group, and 45.4 ± 4.37 years old in the control group. The endoscopic diagnosis of all CRC patients was verified by the histopathologic examination of biopsy specimens. The CRC stage was identified according to TNM/AJCC cancer staging manual (Amin et al., 2017). The UC patients were diagnosed based on established clinical, radiological, endoscopic, and histopathological criteria (Lennard-Jones, 1989; Le Berre et al., 1995). The colonic tissue specimens of the controls were obtained from the healthy colon during colonoscopy performed for benign colonic lesions (e.g. haemorrhoids and solitary rectal ulcer syndrome). Colonic tissue specimens collected from all the participants and stored at - 70 ºC till used. This research was performed according to the ethical guidelines of Helsinki Declaration (World Medical Association Declaration of Helsinki, 2013) and the approval of the study protocol was granted by the Ethics Committee of GISC, Faculty of Medicine, Mansoura University. Prior to study enrolment, forms provided by the GISC ethics committee were signed by all the participants.

*Flow cytometry analysis*

Colonic tissue specimens of all the participants were processed for flow cytometry analysis of CD44 expression, DNA cell cycle, and Annexin V using Accuri C6 Flow Cytometry (Becton Dickinson, Sunnyvale, CA, USA). Analysis of CD44 expression was performed using fluorescent-labeled mouse anti-human CD44 antibody (BD PharmingenTM, PE mouse anti-human CD44) and according to the manufacturer’s protocols. DNA cell cycle was analyzed using Propidium Iodide (Sigma Aldrich, St. Louis, USA). The software was adapted to analyze cell distribution histograms and to estimate the cells in various cell cycle phases using the computer program for mathematical analysis (Dean and Jett, 1974). Annexin-V (BD PharmingenTM, FITC Apoptosis Kit, Cat no 556547, BD Biosciences) was used as one of the most frequently used techniques for detection of apoptosis as well as biochemical and morphological changes in the cells.

*Statistical analysis*

All statistical analyses were done using SPSS 17.0 (Chicago, IL, USA). The Kolmogorov–Smirnov test was used to determine data distribution. The results were expressed as mean ± standard deviation (SD) and frequencies (%). Differences between groups were compared with the Mann–Whitney (U) and Kruskal-Wallis (H) tests. The association between variables was studied using Ranked spearman correlation’s coefficient and linear regression. Receiver operating characteristic (ROC) curve was performed to evaluate the performance of the CD44 marker. All p values were two sided and considered statistically significant at p < 0.05. 

## Results

*Flow cytometry analysis of CD44, DNA cell cycle, and Annexin V/PI staining for apoptosis*

Analysis of CD44 expression revealed significant elevation (p < 0.0001) in both CRC (61.89 ± 9.24) and UC (45.43 ± 7.42) groups in comparison with controls (14.93 ± 4.37). Moreover, a significant difference was found between CRC and UC groups regarding CD44 expression (p < 0.0001). DNA cell cycle of CRC patients evaluated by NDC was abnormally detected in G0/1, S and G2/M (p < 0.0001) but as evaluated by UC patients, the difference was significant in subG1 (p = 0.003), G0/1 and S phase (p < 0.0001). UC group also differed significantly from controls in terms of subG1, G0/1, S phase (p < 0.0001), and G2/M (p = 0.03). Apoptotic cells stained by Annexin V/PI (as apoptotic marker) were distinguished as early (LR) and late (UR) apoptosis (p < 0.0001) in CRC group compared to controls and UC group (LR, p = 0.002 and UR, p = 0.012). However, UC group differed significantly from controls (p < 0.0001) only in terms of late apoptosis (Table 1). 

*Potential relevance of CD44, DNA cell cycle, and Annexin V with the clinicopathological characteristics of CRC patients*

CRC patients were grouped according to tumor site. Accordingly, the tumor was located in the right colon in 16 (36 %) patients (i.e., cecum and ascending colon), in the left colon in 18 (41 %) patients (descending and sigmoid), and in the rectum in 10 (23 %) patients. In CRC group, 8 patients (18 %) were in stage I , 20 patients (46 %) were in stage II (A+B), and 16 patients (36 %) were in stage III (A+B+C). In addition, CRC patients were histopathologically classified according to tumor grade. It was found that 8 patients (18 %) were in grade I , 26 patients (59 %) in grade II , 10 patients (23 %) in grade III . Data revealed by flow cytometry analysis of CD44, DNA cell cycle, and apoptosis by Annexin-V/PI in CRC patients with different clinicopathological tumor characteristics are demonstrated in Table 2. With regard to tumor stage, CD44 was more frequently expressed in stage III (68.06 ± 8.09, p < 0.0001) and stage II (61.96 ± 7.25, p = 0.002) than stage I (50.95 ± 3.66), leading to a significant difference between stage II and III(p = 0.018). With respect to tumor grade, it was found that CD44 expression had extreme elevation in grade III (75.03 ± 4.17) compared to grade II (61.16 ± 7.7, p < 0.0001) and grade I (54.4 ± 6.06, p = 0.002). and between grade I and II (p = 0.02). Nevertheless, no significant difference was found in terms of tumor site. DNA cell cycle was significantly abnormal in stage III compared to stage I (subG1, p < 0.0001 and S phase, p = 0.001) and stage II (S phase, p = 0.003). It also led to a significant difference between stage II and stage I (subG1, p = 0.008). Considering tumor grade, the difference was significant between grade III and I in sub G1, S phase (p = 0.002) and G0/1 (0.037). and between grade III and II the difference was detected only in S phase (p = 0.016). However, regarding tumor site, a significant difference was detected in S phase between right and left colon (p = 0.027) and between left colon and rectum (p = 0.005). Using annexin V/PI, it was found that positive stained cells in early apoptosis (Annexin+ PI-) was higher in stage I than stage III (p < 0.0001) and in stage II was higher than stage III (p = 0.008). However, stage I and II differed only in late apoptosis (p = 0.019). Considering tumor grade, early apoptosis was detected in both grades of III (p = 0.037) and II (p = 0.028) compared to grade I though apoptotic cells in late apoptosis led to a significant difference only between grade III and grade I (p = 0.009) and II (p = 0.042). Regarding tumor site, difference in late apoptosis (Annexin+ PI+) was significant in right (p = 0.003) and left (p = 0.023) colon compared to rectum though no significant difference was found with respect to early apoptosis (Table 2). 

*Relationship between CD44 and DNA cell cycle, Annexin V, and tumor characteristics*

**Figure 1:**
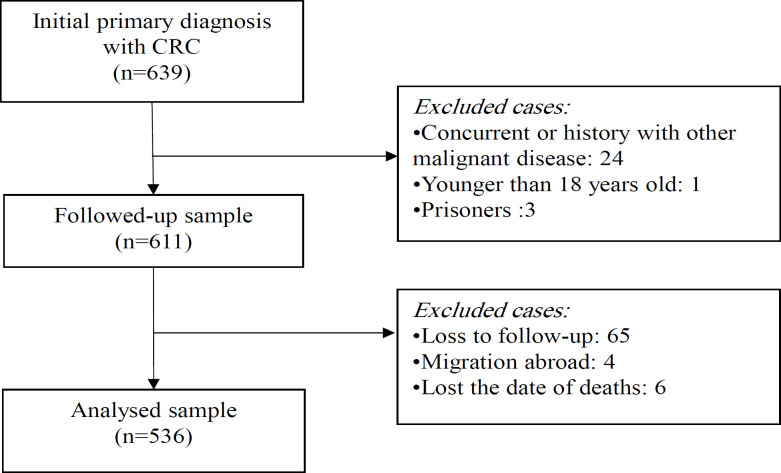
A, CD44 was positively correlated with S phase (rho = 0.396, P = 0.01); B, CD44 was negatively correlated with early apoptosis (rho = - 0.525, P = 0.001)

**Figure 2:**
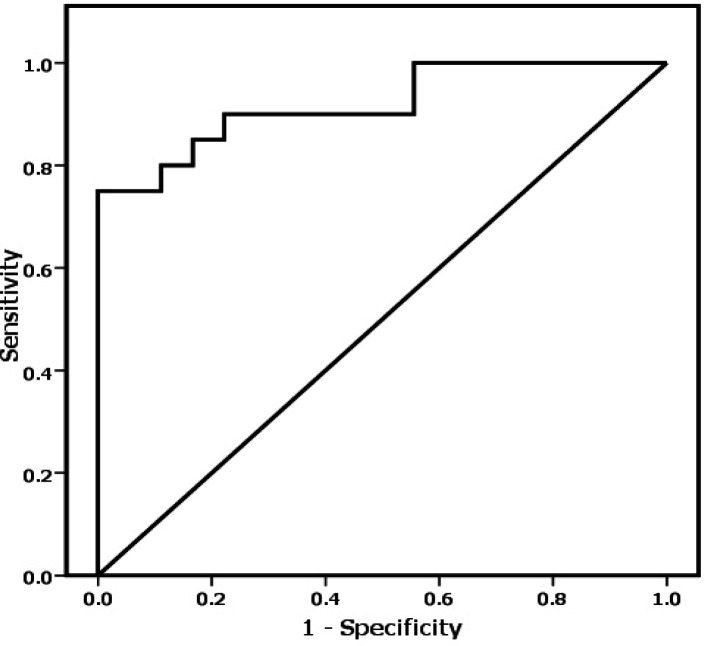
The Receiver Operating Characteristic (ROC) Curve Analysis of CD44 in Colorectal Cancer Patients against Ulcerative Colitis Patients (Area Under ROC; AUROC = 0.919).

Results on the presence of any correlations between CD44 marker, DNA cell cycle, apoptosis, and tumor characteristics in CRC patients are shown in Table 3. CD44 marker was positively associated with S phase (rho = 0.396, p = 0.01), tumor stage, and tumor grade (rho = 0.65, p < 0.0001) but negatively correlated with early apoptosis (rho = - 0.525, p = 0.001) (Figure 1). Based on regression analysis, CD44 marker was as independent predictor (p < 0.0001) for tumor grading and staging in CRC patients, suggesting that CD44 could be used as independent predictor of tumor development (Table 4). Moreover, ROC curve analysis showed high efficacy of CD44 marker (AUROC = 0.919) for discrimination of CRC patients from UC ones (Figure 2). 

**Table 1:** The Levels of CD44, DNA Cell Cycle and Apoptosis of Three Study Groups

Variable	CRC (n = 44)	UC (n = 36)	NDC (n = 30)	P value
CD44	61.89 ± 9.24 *^ #^	45.43 ± 7.42 * º	14.93 ± 4.37^ # ^º	*<0.0001 ^#^<0.0001 º<0.0001
SubG1	15.65 ± 11.65 *	17.33 ± 5.08 * º	12.03 ± 4.93 º	*0.003 º<0.0001
G0/1	54.67 ± 10.68 *^ # ^	64.03 ± 7.91* º	80.35 ± 5.97^ # ^º	*<0.0001 ^#^<0.0001 º<0.0001
S phase	28.27 ± 18.02 *^ # ^	13.66 ± 5.07 * º	3.90 ± 1.04^ #^ º	*<0.0001 ^#^<0.0001 º<0.0001
G2/M	7.02 ± 3.60 ^#^	5.68 ± 4.85 º	2.51 ± 1.54^ # ^º	^#^<0.0001 º0.033
UR	21.55 ± 8.72^#^	16.90 ± 8.12 º	4.94 ± 2.31^# ^º	*0.012 ^#^<0.0001 º<0.0001
LR	9.41 ± 6.17*^#^	4.86 ± 5.56*	1.91 ± 1.29^#^	*0.002 ^#^< 0.0001

**Table 2:** Flow Cytometric Analysis of CD44, DNA Cell Cycle and Apoptosis by Annexin-V/PI in Colorectal Cancer Patients with Different Clinicopathological Tumor Characteristics

Characteristics		CD44	Sub G1	G0/1	S phase	G2/M	Late apoptosis	Early apoptosis
Tumor Stage	Stage I	50.95 ± 3.66º*	28.95 ± 14.97*º	55.5 ± 3.95	13.2 ± 13.84*	6.77 ± 2.76	18.03 ± 4.18º	14.87 ± 2.07*
	Stage II	61.96 ± 7.25º^#^	14.4 ± 9.88º	57.01 ± 8.81	24.11 ± 15.17^#^	6.51 ± 3.29	21.92 ± 7.96º	10.18 ± 6.05^#^
	Stage III	68.06 ± 8.09*^#^	9.63 ± 3.02*	51.19 ± 14.54	42.24 ± 13.99*^#^	7.8 ± 4.43	23.09 ± 11.21	5.28 ± 5.15*^#^
	P Values	* < 0.0001 º 0.02 , ^#^ 0.018	* < 0.0001 º 0.008	NS	* 0.001 ^#^ 0.003	NS	º 0.019	* < 0.0001 ^#^ 0.008
Tumor Grade	Grade I	54.4 ± 6.06*º	13.28 ± 2.93*	61.83 ± 6.27	25.15 ± 6.76*	8.05 ± 3.42	21.15 ± 1.11*	13.93 ± 4.37*º
	Grade II	61.16 ± 7.7^#^º	18.02 ± 13.76	54.4 ± 8.03	24.93 ± 19.64^#^	6.7 ± 3.23	19.68 ± 8.96^#^	8.51 ± 6.15º
	Grade III	75.03 ± 4.17*^#^	8.53 ± 1.32*	46.17 ± 16.68	46.9 ± 6.85*^#^	6.96 ± 5.51	30.17 ± 9.12*^#^	7.27 ± 6.22*
	P values	* 0.002, º 0.022 ^#^ < 0.0001	*0.002	NS	* 0.002 ^#^ 0.016	NS	* 0.009 ^#^ 0.042	* 0.037 o 0.028
Tumor Site	Rt colon	63.2 ± 11.05	11.95 ± 5.7	50.68 ± 13.47	32.19 ± 15.65º	5.91 ± 4.15	24.86 ± 8.52*	9.71 ± 6.12
	Lt colon	62.69 ± 6.46	21.3 ± 14.78^#^	57.09 ± 7.75	19.16 ± 17.41^#^º	7.36 ± 3.18	22.61 ± 4.83^#^	9.49 ± 7.3
	Rectum	55.9 ± 10.36	8.7 ± 1.6^#^	58.03 ± 7.53	45.2 ± 8.96^#^	8.97 ± 2.46	9.57 ± 9.22*^#^	8.33 ± 1.39
	P values	NS	^#^ 0.045	NS	º 0.027 ^#^ 0.005	NS	* 0.003 ^#^ 0.023	NS

* ^#^ º, A statistically significant difference at P < 0.05 was shown between each marked pair of tumor characteristics. NS, not significant.

**Table 3:** Spearman's Correlation (rho) between CD44 with Tumor Characteristics, S phase of DNA Cell Cycle and Apoptosis by Annexin V/PI in Colorectal Cancer (CRC) Patients

Characteristics	S phase	Late apoptosis	Early apoptosis	Tumor stage	Tumor grade
rho	0.396	0.56	-0.525	0.655	0.652
P	< 0.01*	< 0.0001**	< 0.001**	< 0.0001**	<0 .0001**

*, Correlation is significant at the 0.05 level (2-tailed), **, Correlation is significant at the 0.01 level (2-tailed).

**Table 4:** Regression Analysis of CD44, Proliferative Activity of DNA Cell Cycle (S phase) and Apoptosis by Annexin-V/PI as Independent Predictors in Tumor Staging and Grading of Colorectal Cancer (CRC) Patients

Independent predictor	Tumor Staging				Tumor grading			
	R	R^2^	B	P	R	R^2^	B	P
CD44	0.656	0.43	0.052	< 0.0001**	0.645	0.416	0.042	< 0.0001**
S phase	0.612	0.374	0.025	< 0.0001**	0.571	0.326	0.107	0.011*
UR	0.199	0.039	0.017	0.219	0.270	0.073	0.019	0.092
LR	0.573	0.328	-0.068	< 0.0001**	0.341	0.116	-0.033	0.031*

*, Correlation is significant at the 0.05 level (2-tailed).

## Discussion

CD44 marker is a cell-surface glycoprotein involved in cell-cell interaction, adhesion, and migration (Park et al., 2021). CD44 marker participates in multiple physiological processes and its aberrant expression and dysregulation contributes to tumor initiation and progression (Xu et al., 2020). Previous studies on the colon found that CD44 pattern of staining was mainly membranous (Al-Maghrabi et al., 2012; Holah et al., 2017) and there was a highly significant difference between carcinoma cases and normal cases (p = 0.02) (Chai et al., 2013), which is in line with our findings with respect to CD44 expression. According to a previous study, there was a significant difference between CRC cases and studied adenoma in terms of CD44 expression (Zavrides et al., 2005). Similarly, we observed a significant diference between CRC and UC patients in this regard. Zavrides et al., (2005) in their study followed up CRC patients for five years. They found that expression of CD44 was not related to patient sex and age but to tumor differentiation, stage, and site. Some previous research reported that CD44 expression level was higher in high-grade CRC compared with low-grade tumors, and this overexpression was associated with reduced patient survival (Ropponen et al., 1998). In line with aforementioned study, we found up-regulation of CD44 in high-grade CRC (grade III) compared to low-grade CRC(II and I). On the contrary, Ylagan et al., (2000) reported that the loss rather than CD44 increased expression was associated with increased tumor aggressiveness. Similarly, Dallas et al., (2012) reported that down regulation of CD44 led to an increase in the metastatic and migratory potential of CRC cells. This inconsistency in findings can be due to alternative splicing of the CD44 pre-RNA. Treatment of CRC, which is rarely diagnosed at early stages, is largely based on the stage of the cancer (Frank et al., 2010). It has been shown that in lymphomas, as well as in gastric and cervical carcinomas, high CD44 expression is correlated with more advanced tumor stage and possibly with poor prognosis (Carr et al., 2002). In line with Zhao et al.’ study (2015), CD44 higher expression was also detected in the current study , which was correlated with advanced tumor stage. However, Holah et al., (2017) found no significant relation between CD44 expression and grade or stage of tumor. Additionally, a statistically significant association was detected between CD44 positive expression and left-sided tumor in previous studies (Holah et al., 2017; Michl et al., 2015). It was also found that left sided localization of CRC had a better prognosis compared with right sided localization of CRC (Meguid et al., 2008). No significant association was recorded in the current study between CD44 and tumor site, which could be due to the difference in number and type of Egyptian patients. Flow cytometry can identify cases with high cell proliferation (high S phase and G2/M) which are very susceptible to different mutagenic agents or chemicals, resulting in neoplastic transformation (Attallah et al., 2009). DNA cell cycle of CRC patients in the current study revealed a significant frequency of cells in S phase (p < 0.0001) compared to UC and control groups. Higher proliferative activity of CRC (high S phase) was related to higher staging (stage III) and grading (grade III and grade II) and also correlated with CD44 overexpression. Similarly, Fernandez et al., (2004) reported that CD44 expression level was related to proliferation in CRC but not to patient outcome. CD44 + cells had less spontaneous apoptosis and were more resistant to drug-induced cell death (Wang et al., 2012). Accordingly, CD44 up-regulation in the current study was correlated negatively with early apoptosis detected by Annexin V/PI in CRC patients, suggesting that CD44 expression may inhibit apoptosis probably by inducing anti-apoptotic effect . In line with our findings, Madjd et al., showed that CD44+ cells led to higher expression of anti-apoptotic protein bcl-2 in breast cancer cells (Madjd et al., 2009). In conclusion, CD44 elevated expression was significantly associated with higher proliferative activity of CRC and negatively correlated with apoptosis, suggesting that CD44 induced by malignant cells might down regulate apoptosis to the tumor cells and subsequently leading to decline of growth inhibition and increase of the ability to resist chemotherapy. Therefore, CD44 can be used as an independent predictor for tumor development and as effective therapeutic target. However, further studies regarding the role of CD44 marker in the CRC metastasis and tumor recurrence after treatment are needed.

## Author Contribution Statement

All authors confirm contribution to the manuscript as follows: HI and HE study conception and design, writing original draft. ME medical history and tissue samples. HE, DH and HI methodology and collection of data. HE, ME and HI, analysis and interpretation of results. All authors approved the final version of the manuscript.

## Ethics

This study was part of a MSc thesis and its protocol was approved by the Ethics Committee of GISC, Faculty of Medicine, Mansoura University, Mansoura, Egypt. 

## Availability of Data

The original data is available on request from the corresponding author or HE (email: elemshaty_h@yahoo.com).

## Conflicts of interest

All the authors declare that they have no conflict of interest.
